# P-952. Optimizing Oral Antimicrobial Therapy at Hospital Discharge: Impact of Antimicrobial Stewardship Pharmacist vs. Team-Based Intervention

**DOI:** 10.1093/ofid/ofaf695.1154

**Published:** 2026-01-11

**Authors:** Justin Siegfried, Kassandra Marsh, Yihan Li, Calvin Chan, Quy Huynh, Dana Mazo, Yanina Dubrovskaya

**Affiliations:** NYU Langone Health, New York, New York; NYU Langone Health, New York, New York; NYU Langone Health, New York, New York; NYU Langone Health, New York, New York; NYU Langone Health, New York, New York; New York University, New York, NY; NYU Langone Health, New York, New York

## Abstract

**Background:**

Implementing a formal program for the review of oral antibiotic discharge prescriptions (AbxRx) is often limited by staffing constraints. At our institution, primary teams can choose to reach out to an antimicrobial stewardship pharmacist (ASP) for guidance on AbxRx at discharge via curbside consultation (DCC). We described DCC and compared clinical outcomes between ASP vs. team-based (TB) groups.

**Figure:**
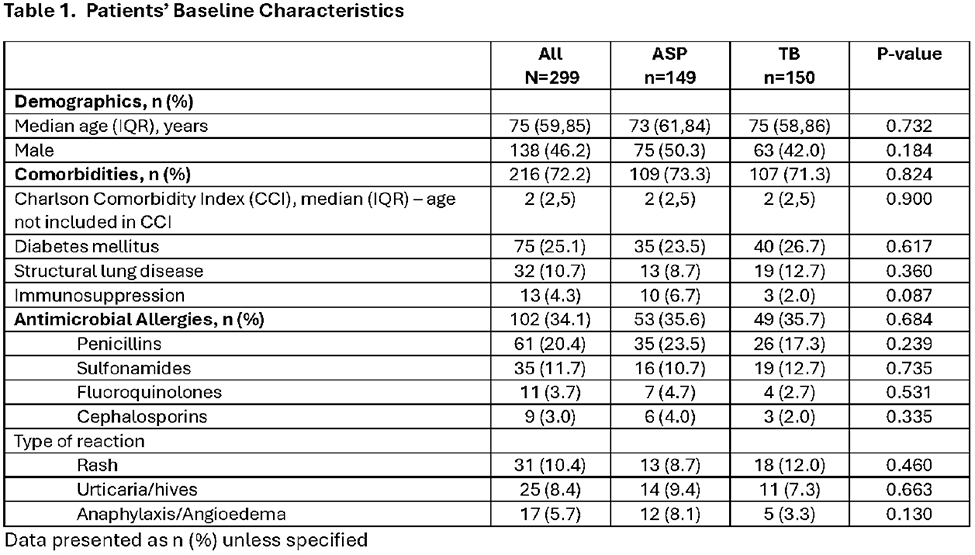

**Figure:**
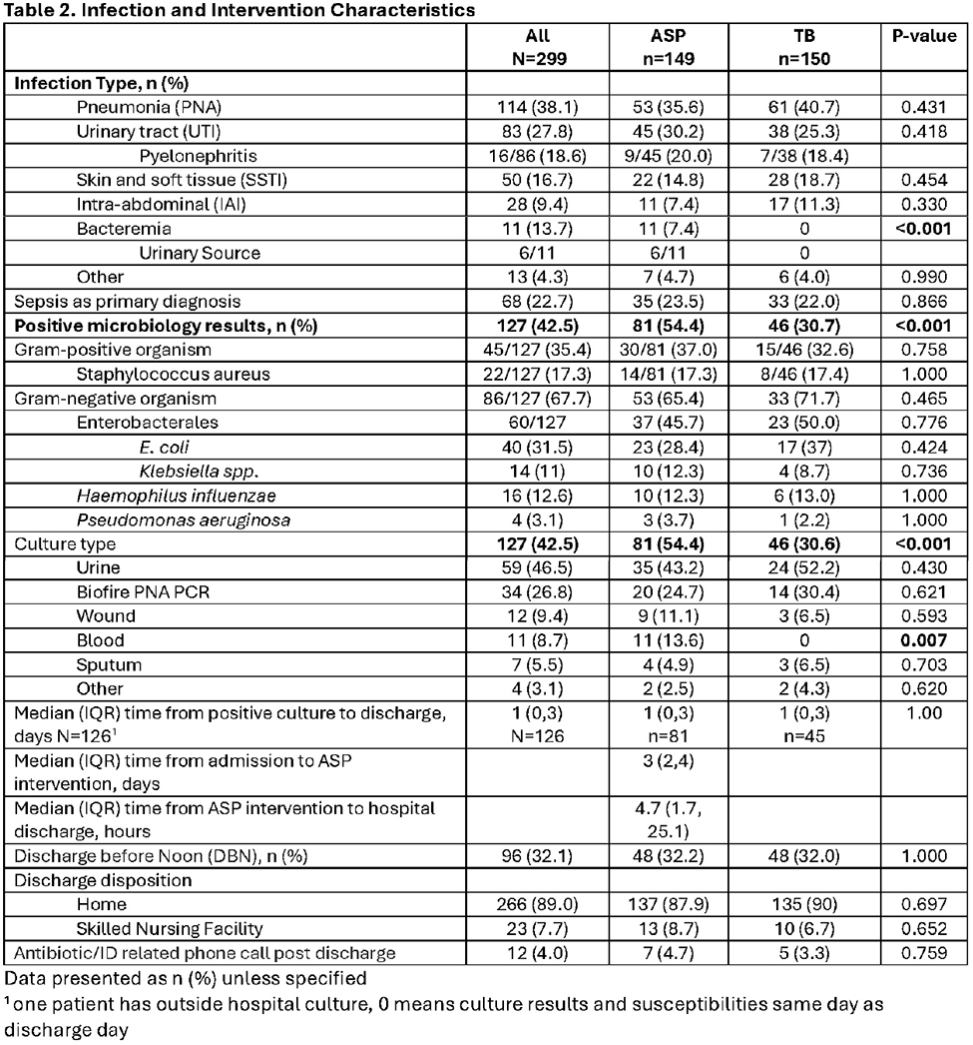

**Methods:**

This was a retrospective study of adult inpatients who received an AbxRx between 1/2024 to 6/2024 with or without ASP intervention. TB group consisted of a medicine (MD/DO) attending and an advanced practice provider (APP) without ASP intervention. The primary composite outcome was the appropriateness of AbxRx, defined as use of narrow spectrum of activity, dosing and duration consistent with local ASP guidelines. Secondary outcomes include length of stay (LOS), readmission, and incidence of Clostridiodies difficile infection (CDI).

**Figure:**
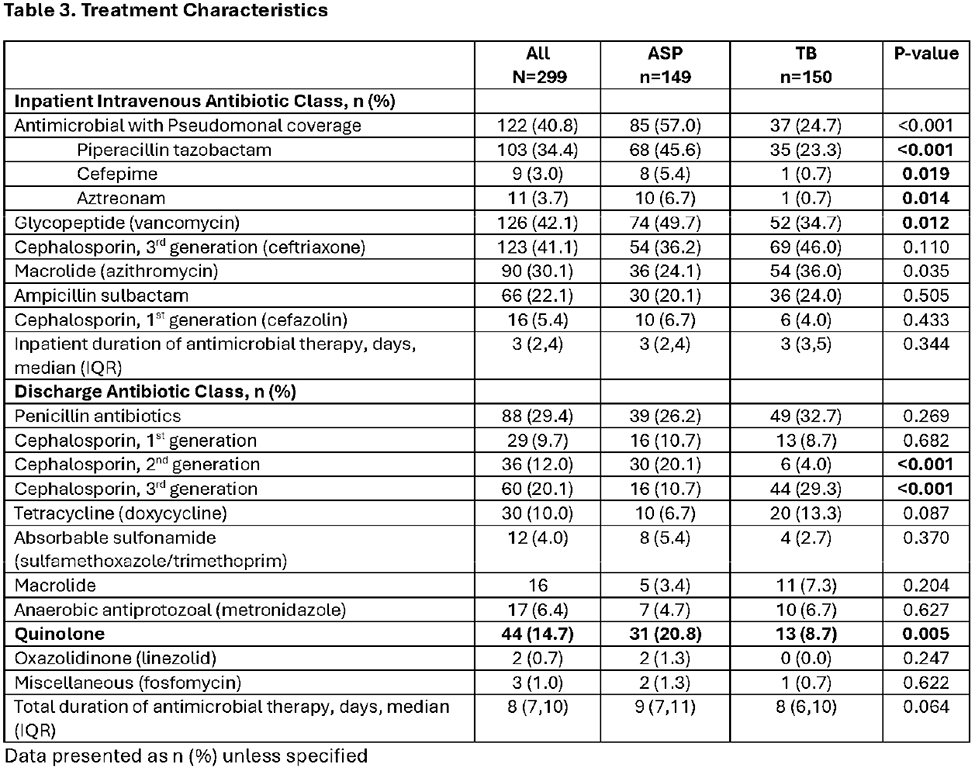

**Figure:**
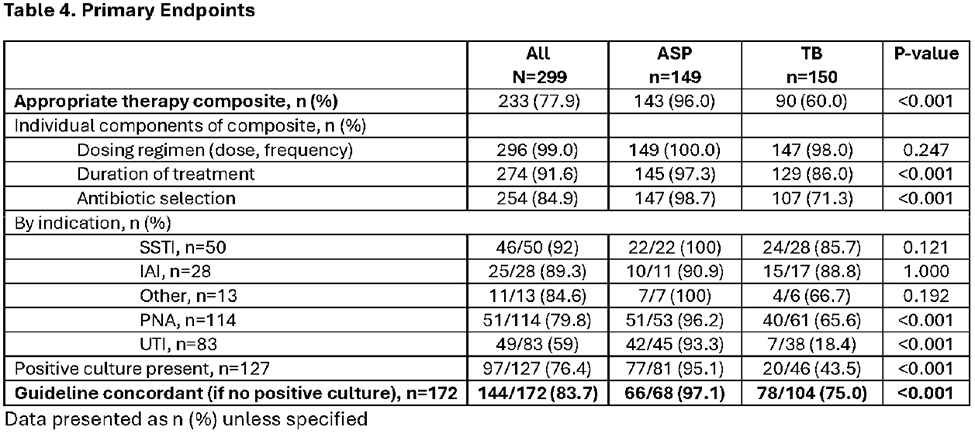

**Results:**

Of 359 reviewed, 299 patients prescribed an AbxRx were included (ASP n=150, TB n=149). Age and Charlson comorbidity index were similar between groups. The most common infection was pneumonia (38%), urinary tract (27.8%), and skin soft tissue infections (16.7%). APPs were more likely to initiate DCC (64.4% vs 19.5% attending). DCC were triggered by bacteremia (13.7% vs 0%, P=< 0.001), intravenous abxRx with Pseudomonal coverage (69.1% vs 40.7%, P< 0.001) and presence of positive cultures (54.4% vs 30.7%, P< 0.001). Median time from admission to ASP intervention was 3 days (IQR 2-4), from ASP intervention to discharge was 4.7h (IQR 1.7-25.1h). More patients in the ASP group were discharged on cefuroxime (20.1% vs. 4%, P< 0.001) while more patients in TB group were discharged on cefpodoxime (10.7% vs. 29.3%, P< 0.001). Patients in the ASP group were more likely to have appropriate AbxRx (96% vs. 60%, P< 0.001) and higher adherence to AS guidelines (97.1% vs. 75%, P< 0.001). LOS (3 vs. 3 days, P=0.34), readmission (4.7% vs. 2%, P=0.22) and CDI (0%) were similar between groups.

**Conclusion:**

DCC were more commonly triggered by APPs, likely for the need of higher-level infectious disease training and comfortability in interpreting cultures. ASP improved the appropriateness of AbxRx with increased narrow spectrum antibiotic use and adherence to AS guidelines.

**Disclosures:**

All Authors: No reported disclosures

